# A phase Ib randomized multicenter trial of isolated hepatic perfusion in combination with ipilimumab and nivolumab for uveal melanoma metastases (SCANDIUM II trial)☆

**DOI:** 10.1016/j.esmoop.2024.103623

**Published:** 2024-07-02

**Authors:** R. Olofsson Bagge, A. Nelson, A. Shafazand, C. Cahlin, A. Carneiro, H. Helgadottir, M. Levin, M. Rizell, G. Ullenhag, S. Wirén, P. Lindnér, J.A. Nilsson, L. Ny

**Affiliations:** 1 Department of Surgery, Institute of Clinical Sciences, Sahlgrenska Academy, University of Gothenburg, Gothenburg; 2 Department of Surgery, Sahlgrenska University Hospital, Gothenburg; 3 Wallenberg Centre for Molecular and Translational Medicine, University of Gothenburg, Gothenburg; 4 Department of Oncology, Institute of Clinical Sciences, Sahlgrenska Academy at University of Gothenburg, Sahlgrenska University Hospital, Gothenburg; 5 Department of Oncology, Sahlgrenska University Hospital, Gothenburg; 6 Transplant Institute, Institute of Clinical Sciences, Sahlgrenska Academy at University of Gothenburg, Sahlgrenska University Hospital, Gothenburg; 7 Department of Haematology, Oncology and Radiation Physics, Skåne University Hospital Comprehensive Cancer Center, Lund; 8 Department of Oncology, Karolinska University Hospital, Stockholm; 9 Department of Radiology, Immunology, Genetics, and Pathology, Uppsala University, Uppsala; 10 Department of Radiation Sciences, Umeå University Hospital, Umeå, Sweden; 11 Harry Perkins Institute of Medical Research, University of Western Australia, Perth, Australia

**Keywords:** isolated hepatic perfusion, immune checkpoint inhibitor, PD-1, CTLA-4, liver metastases, uveal melanoma

## Abstract

**Background:**

Uveal melanoma (UM) is a rare malignancy where 50% of patients develop metastatic disease primarily affecting the liver. Approximately 40% of patients with metastatic UM respond to one-time isolated hepatic perfusion (IHP) with high-dose melphalan. This phase I trial investigates the safety and clinical efficacy of IHP combined with ipilimumab (IPI) and nivolumab (NIVO).

**Patients and methods:**

Immunotherapy-naïve patients were randomized in this phase I trial to receive either IHP followed by IPI 3 mg/kg and NIVO 1 mg/kg (IPI3/NIVO1) for four cycles (post-operative arm), or one cycle of preoperative IPI3/NIVO1, IHP and then three cycles of IPI3/NIVO1 (pre-post-operative arm), followed by maintenance therapy with NIVO 480 mg for 1 year.

**Results:**

Eighteen patients were enrolled and randomized. Three patients did not undergo IHP as planned. In total, 11/18 patients (6 in the post-operative arm and 5 in the pre-post-operative arm) did not complete the planned four cycles of IPI3/NIVO1. Toxicity to IHP was similar in both groups, but the number of immune-related adverse events (AEs) was higher in the pre-post-operative arm. Among assessable patients, overall response rate was 57% in the post-operative arm (4/7) and 22% in the pre-post-operative arm (2/9).

**Conclusions:**

Combination therapy with IHP and IPI3/NIVO1 was associated with severe AEs. The efficacy of this combination is encouraging with high response rates. One cycle of preoperative IPI/NIVO before IHP did not show potential benefits in terms of safety or efficacy.

## Highlights

• Patients with liver metastases of UM have limited treatment options that only moderately prolong survival.
• The combination of IHP using melphalan and IPI3/NIVO1 is safe but with significant toxicity.
• One cycle of preoperative IPI/NIVO does not seem beneficial in this setting.
• The treatment combination has encouraging efficacy that warrants further evaluation in a phase II study.

## Introduction

Uveal melanoma (UM) is a rare disease accounting for ∼3% of all melanomas. Despite effective treatment of the primary tumor, around 50% of patients develop metastases.^1^ The organ distribution of metastasis is strongly hepatotropic, with isolated liver metastases observed in over half of the patients with metastatic disease. In this situation, patients have a poor prognosis. The median survival is 4-10 months, and very few patients survive >5 years.^1^^,^^2^

Treatment with immune checkpoint inhibitors (ICIs) has limited clinical efficacy in patients with metastatic UM. Ipilimumab (IPI) combined with nivolumab (NIVO) has overall response rates (ORRs) ranging from 10% to 18% and an uncertain impact on overall survival (OS).^3^^,^^4^ The combination of pembrolizumab and epigenetic therapy with a histone deacetylase inhibitor has resulted in durable responses in a subset of patients.^5^ A randomized phase III trial using tebentafusp, a bispecific fusion protein linking melanoma cells with T cells, in human leukocyte antigen (HLA)-A∗02:01 serotype patients with metastatic UM demonstrated a significant improvement in OS (22 versus 16 months).^6^ These trials show that durable responses to immunotherapy are possible to obtain in some patients, but a high unmet clinical need still exists.

Isolated hepatic perfusion (IHP) with melphalan is a regional treatment where the liver is completely isolated from the systemic circulation, allowing for the delivery of a high concentration of a chemotherapeutic agent with minimal systemic exposure.^7^^,^^8^ In this treatment the effective dose exposed to the liver parenchyma is ∼10 times higher than the dosing used during myeloablative treatment with melphalan in hematological malignancies. In a randomized phase III trial (SCANDIUM), we recently reported a superior ORR (40% versus 4%) and a 4-month benefit in progression-free survival (PFS; 7.4 versus 3.3 months) with IHP compared to best alternative care in treatment-naïve patients with isolated liver metastases of UM.^9^

While apoptosis classically has been regarded as a silent type of cell death, there is increasing evidence that certain types of chemotherapeutic agents trigger a type of cell death associated with inflammatory signaling and activation of immunity, i.e. an immunogenic cell death.^10^ For example, anthracyclines cause an immunogenic type of apoptosis, with hallmarks that include surface translocation of calreticulin, serving as an ‘eat me’ signal for phagocytes, and also release of high-mobility group box 1 protein triggering an inflammatory immune response, leading to the engulfment of apoptotic bodies by dendritic cells (DCs) and the activation of cytotoxic CD8+ T cells.^11^ We have previously shown that upon exposure to melphalan, melanoma cells increased the expression of immune-related markers, including programmed death-ligand 1, major histocompatibility complex class I and Hsp70. When peripheral blood mononuclear cells (PBMCs) were co-cultured with these melanoma cells pre-exposed to melphalan, this triggered an expansion of CD33+CD14+CD16+ non-classical monocytes as well as CD8+ T cells in the co-cultured PBMCs.^12^ The combination of melphalan with adoptive cell therapy also significantly prolongs survival in mice with advanced B-cell lymphomas or colorectal cancer, where melphalan enhances immune response by increasing cytokine release, DC activation and the potentiation of both endogenous CD8+ T cells and adoptively transferred tumor-specific CD4+ T cells.^13^

The hypothesis behind the present trial was that IHP, beyond providing a direct antitumoral effect, may also activate the immune system through these immunological mechanisms, and that this could be potentiated combining the treatment with ICIs. This is further supported by early clinical data showing that after isolated limb perfusion (ILP) for melanoma there is an increase in tumor-specific CD8+ T cells in blood after 1 month.^14^ Another study showed that melanoma patients with certain immunological profiles, including higher expression of HLA-DR on CD8+ T cells as well as a higher fraction of memory CD45RO+ CD8+ T cells, were more likely to respond favorably to isolated limb perfusion, indicating that T cells are important in the clinical effect of this treatment.^15^ Furthermore, in patients with UM metastases, higher levels of CD8+ T-cell tumor infiltration correlate positively with survival after IHP.^16^ Recent evidence has also shown the benefit of a neoadjuvant approach with ICIs in patients with cutaneous melanoma.^17^ However, the impact of ICI timing in the setting of IHP is unknown, which warrants a comparison of a post-operative versus a pre-post-operative approach.

This phase I trial (SCANDIUM II) investigates the safety, tolerability and timing of IHP combined with IPI and NIVO as a treatment for patients with liver-dominant metastases of UM. In this pre-planned analysis, we report the primary endpoints of safety and clinical efficacy, including ORR and PFS.

## Patients and methods

### Patients

Patients with histologically or cytologically confirmed liver metastases from UM were eligible if they had measurable disease according to Response Evaluation Criteria in Solid Tumors version 1.1 (RECIST 1.1) criteria^18^ and had liver-dominant disease as assessed by the investigator. Prior treatment with IHP or immunotherapy was not allowed but previous chemotherapy and/or regional therapy other than perfusion therapy was accepted. Patients were excluded if the tumor volume occupied 50% or more of the liver as assessed by computed tomography (CT) or magnetic resonance imaging (MRI); if the patient had a history of significant autoimmune disease; if there was significant heart, lung or renal dysfunction; or if the patient had a body mass index >35 kg/m^2^.

### Study design and treatment

**Figure 1:**
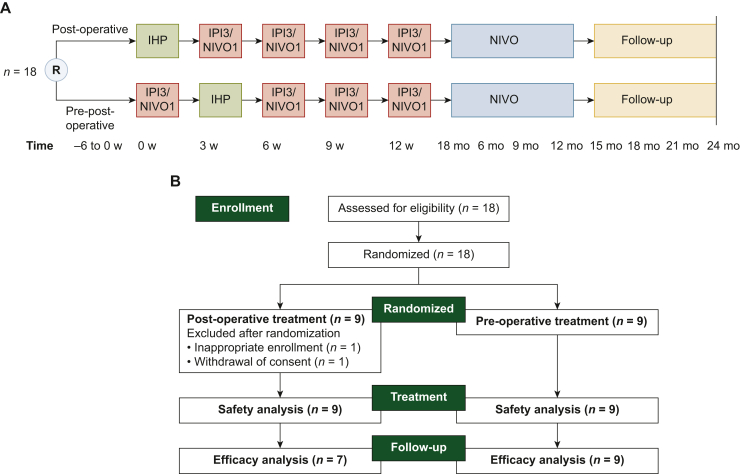
**Study design and CONSORT diagram.** (A) Treatment outline and (B) CONSORT diagram. CONSORT, Consolidated Standards of Reporting Trials; IHP, isolated hepatic perfusion; IPI3/NIVO1, ipilimumab 3 mg/kg and nivolumab 1 mg/kg; NIVO, nivolumab 480 mg fixed dose; mo, months; R, randomization; w, weeks. Created with BioRender.com.

This was a prospective, multicenter, open-label, phase Ib trial randomizing patients in a 1 : 1 ratio to receive either IHP followed by combination immunotherapy with four cycles of IPI 3 mg/kg and NIVO 1 mg/kg (IPI3/NIVO1) every 3 weeks (post-operative arm) or one preoperative cycle of IPI3/NIVO1 before IHP followed by three cycles of IPI3/NIVO1 (pre-post-operative arm) (Figure 1A). Thereafter, both arms received monotherapy with NIVO 480 mg every 4 weeks for up to 1 year.

IHP was carried out at Sahlgrenska University Hospital, Gothenburg, Sweden. The procedure has previously been described in detail.^8^ In summary, a shunt was inserted into the iliac and jugular veins and connected to an external centrifugal pump shunting blood from the lower extremity after clamping the inferior vena cava. The vena cava was isolated infrahepatically above the renal veins and suprahepatically between the liver and the diaphragm. A catheter was placed in the retrohepatic portion of the vena cava for perfusion outflow and the hepatic artery proper was cannulated for perfusion inflow. The portal vein was clamped, and the catheters were connected to a perfusion system. Perfusion was carried out with a target flow rate of 500-1200 ml/min with a target liver temperature of 40°C. When steady-state conditions in the perfusion circuit were established, melphalan at a dose of 1 mg/kg body weight was added to the perfusion system and was divided into two doses given 30 min apart. Perfusion was continued for 60 min, then discontinued and the liver was irrigated (Ringer’s acetate, Baxter, Kista, Sweden). The shunts and the perfusion circuit were disconnected, and the catheters were removed.

### Study assessments

Clinical and radiological evaluation was carried out every 3 months up to 2 years by either CT or MRI of the liver using the same modality as at the baseline examination, with additional CT imaging of the thorax. Laboratory values and vital signs were assessed regularly. After IHP, patients were admitted to a post-operative unit overnight and then transferred to a surgical ward. Patients were followed according to routine clinical care procedures, with a length of stay of ∼5-7 days if no complications evolved. Treatment and follow-up with ICI was carried out according to local clinical routine, with clinical examinations and laboratory analysis the first day in each treatment cycle as a minimum. Safety was assessed by collecting data for adverse events (AEs), which were graded according to the National Cancer Institute Common Terminology Criteria for Adverse Events (CTCAE) version 4.0.

### Endpoints

The primary endpoint was to determine the incidence and severity of AEs and serious adverse events (SAEs). The secondary endpoints were ORR, clinical benefit rate (CBR), duration of response (DOR), PFS and OS (data immature at data cut-off and are not presented in this report). AEs were registered from the first treatment dose until 100 days after the last treatment or 30 days after the last dose if the participant started a new anticancer therapy, whichever occurred first. Response was graded according to RECIST 1.1.^18^ ORR was defined as the percentage of patients with a best overall response of complete response (CR) or partial response (PR). CBR was defined as the percentage of patients with a best overall response of CR, PR or stable disease (SD). DOR was defined as the time from response to documented progression at any site, or death from any cause. PFS was defined as the time from randomization to documented progression at any site, or death from any cause according to RECIST 1.1 criteria. Efficacy was evaluated in assessable patients in the overall population.

### Statistical analysis

The primary aim of this trial was to determine safety of the combination of IHP using melphalan and IPI3/NIVO1. The safety of both these treatments on its own is well known, but the safety of the combined treatment is completely unknown. The number of patients needed to establish the safety of the treatment combination was discussed thoroughly with regulatory authorities, the Swedish Medical Product Agency.

With the intent to balance the risk for potential unknown AEs and potential benefits in patients with a rare cancer disease, a sample size of 9 patients in each arm (18 patients in total) was judged reasonable. Randomization using variable block size was carried out using the MediCase eCRF system. Time-to-event analysis was carried out using the Kaplan–Meier methodology and is reported using medians together with 95% confidence intervals (CIs). Fisher’s exact test was used to compare ORR.

### Study oversight

This was an investigator-initiated study with the support of free study drugs (IPI and NIVO) from BMS, CA184-587. The original protocol and all amendments were approved by the Swedish Medical Products Agency (EudraCT number 2020-003188-24) and the Swedish Ethical Review Authority (Dnr 2021-05391-02) and was registered at ClinicalTrials.gov (NCT04463368). The study was conducted in accordance with the protocol, Good Clinical Practice guidelines and the provisions of the Declaration of Helsinki. All patients provided written informed consent before inclusion in the trial. All AEs and SAEs were prospectively and periodically monitored by an independent data monitoring board with the authority to recommend termination of the study based on safety concern. No hard stopping criteria were used.

## Results

### Patients and treatments

From May 2021 to August 2022, 18 patients were screened, and 18 patients were enrolled at six sites in Sweden (Figure 1B). The patients were randomly assigned to the post-operative arm (nine patients) or the pre-post-operative arm (nine patients). The demographics and baseline disease characteristics of the patients are described in Table 1. The randomization resulted in the pre-post-operative arm having more females, higher number of patients who had received prior systemic therapy and presence of extrahepatic disease than in the post-operative arm. The median duration of follow-up at the time of data cut-off for this analysis (30 June 2023) was 19.1 months (range 11.0-25.6 months). After treatment initiation, it was found that one patient in the post-operative arm did not fulfill the criteria for measurable disease according to RECIST 1.1. This patient was evaluated for safety but not for efficacy. As seen below, in the ‘Safety’ section, one patient in the post-operative arm withdrew from the trial without receiving any treatment and before radiological evaluation; this patient was not included in the efficacy evaluation. Consequently, 7/9 patients in the post-operative arm and 9/9 patients in the pre-post-operative arm were included in the efficacy analysis.

**Table 1:** Baseline patient characteristics

	Post-operative (*n* = 9)	Pre-post-operative (*n* = 9)
Age, median (range), years	62 (48-73)	65 (54-70)
Sex, *n* (%)		
Female	1 (11)	5 (56)
Male	8 (89)	4 (44)
ECOG PS, *n* (%)		
0	9 (100)	8 (89)
1	0 (0)	1 (11)
Previous treatment for metastatic disease, *n* (%)		
No previous treatment	9 (100)	5 (56)
Chemotherapy^a^	0	3 (33)
Surgical resection	0	1 (11)
Radiation therapy	0	1 (11)
Largest metastatic lesion, *n* (%)		
≤3.0 cm	6 (67)	7 (78)
3.1-8.0 cm	3 (33)	0 (0)
≥8.1 cm	0 (0)	2 (22)
Mean tumor volume, % (range %)	11 (1-45)	11 (5-40)
Metastatic sites, *n* (%)		
Isolated liver metastases	8 (89)	5 (56)
Liver and extrahepatic metastases	1 (11)	4 (44)
Years since primary diagnosis, median (range)	0.9 (0.2-6.8)	2.2 (0.1-10.9)
Lactate dehydrogenase >ULN, *n* (%)	5 (56)	5 (56)

ECOG PS, Eastern Cooperative Oncology Group performance status; IQR, interquartile range; ULN, upper limit of normal.

a Temozolomide.

Eight of nine patients (89%) in the post-operative arm and 7/9 patients (78%) in the pre-post-operative arm received IHP as per protocol. The median IHP operating time was 5.5 h (range 4.5-10.3 h) in the post-operative arm and 5.8 h (range 4.5-7.0 h) in the pre-post-operative arm. The median inpatient stay time was 8 days (range 5-11 days) in the post-operative arm and 9 days (range 7-14 days) in the pre-post-operative arm. The median perioperative liver temperature was 40.2°C (range 39.7-40.7°C), the median perfusion flow rate was 600 ml/min (range 50-1300 ml/min) and the median blood loss (including waste) was 775 ml (range 100-2100 ml). Values were similar across the two arms (not shown). A peroperative assessment of liver tumor involvement was conducted, showing that the median tumor volume was 5% of the liver volume (range 1%-45%) in the pre-post-operative group and 5% (range 5%-40%) in the post-operative group.

In the post-operative arm, patients received a median of 3 cycles (range 1-4 cycles) of IPI3/NIVO1; 3/9 patients (33%) received all four cycles of IPI3/NIVO1. In the pre-post-operative arm, patients received a median of 3 cycles (range 1-4 cycles) of IPI3/NIVO1; all patients (9/9) received one cycle of pre-post-operative IPI3/NIVO1 and 3/9 patients (33%) completed all three cycles of post-operative IPI3/NIVO1.

### Safety

The primary endpoint of the trial was safety for the combined treatment of IHP and IPI3/NIVO1. Among patients in the post-operative arm, 8/9 patients (89%) had at least one grade 3 or 4 AE that was deemed by the investigators to be related to IHP (Table 2). The most frequent grade 1 or 2 events were fever and anemia that were related to IHP (Supplementary Table S1, available at https://doi.org/10.1016/j.esmoop.2024.103623). One patient (11%) in the post-operative arm did not receive IHP due to intraoperative damage to the hepatic artery. This AE resolved without sequelae within 4 weeks, but the patient chose to withdraw from the trial and received treatment outside of this trial. Among the seven patients in the pre-post-operative arm who underwent IHP, five patients (71%) had at least one grade 3 or 4 AE that was deemed by the investigators to be related to IHP. One patient did not receive IHP due to a perioperative observation that >50% of the liver was occupied with metastases and one patient did not receive IHP due to grade 3 encephalitis following one cycle of pre-post-operative IPI3/NIVO1. This latter patient received IHP outside of the trial 5 months later.

**Table 2:** Frequency of patients with grade 3-4 adverse events attributed by the treating physician to IHP or IPI and NIVO graded according to CTCAE 4.0 and classified according to MedDRA

Event^a^	Post-operative arm		Pre-post-operative arm	
**Event attributed to preoperative IPI/NIVO**	**Grade 3**	**Grade 4**	**Grade 3**	**Grade 4**
Immune system disorders				
Encephalitis	N/A	N/A	1/9	0
General disorders and administration site conditions				
Pyrexia	N/A	N/A	1/9	0
**Event attributed to IHP**	**Grade 3**	**Grade 4**	**Grade 3**	**Grade 4**
Blood and lymphatic system disorders				
Anemia	1/9	0	2/7	0
Leukopenia	0	0	1/7	0
Cardiac disorders				
Syncope	1/9	0	0	0
General disorders and administration site conditions				
Pyrexia	0	0	1/7	0
Hepatobiliary disorders				
Hepatic infection bacterial	1/9	0	0	0
Injury, poisoning and procedural complications				
Artery dissection	1/9	0	0	0
Intraoperative arterial injury	0	1/9	0	0
Procedural complication^b^	3/9	0	3/7	0
Investigations				
Alanine aminotransferase increased	5/9	1/9	3/7	0
Aspartate aminotransferase increased	5/9	1/9	2/7	1/7
Blood bilirubin increased	0	0	1/7	0
C-reactive protein increased	1/9	0	2/7	0
Renal and urinary disorders				
Acute kidney injury	0	0	1/7	0
Nephritis	0	0	1/7	0
Respiratory, thoracic and mediastinal disorders				
Pleural effusion	0	0	1/7	0
Pneumonia	0	0	1/7	0
Pulmonary embolism	0	0	1/7	0
Vascular disorders				
Arterial thrombosis	1/9	0	0	0
Hypertension	0	0	1/7	0
**Event attributed to post-operative IPI/NIVO**	**Grade 3**	**Grade 4**	**Grade 3**	**Grade 4**
Blood and lymphatic system disorders				
Autoimmune hemolytic anemia	0	1/8	0	0
Thrombocytopenia	1/8	0	0	0
Endocrine disorders				
Diabetes mellitus	0	0	0	1/8
Gastrointestinal disorders				
Colitis	1/8	0	0	0
Immune system disorders				
Encephalitis	1/8	0	0	0
Investigations				
Alanine aminotransferase increased	1/8	0	2/8	0
Aspartate aminotransferase increased	1/8	0	2/8	0
Blood alkaline phosphatase increased	0	0	1/8	0
Blood bilirubin increased	1/8	0	0	0
Nutrition				
Decreased appetite	1/8	0	0	0
Renal and urinary disorders				
Nephritis	0	0	1/8	0
Respiratory, thoracic and mediastinal disorders				
Pneumonitis	0	0	1/8	0

CTCAE, Common Terminology Criteria for Adverse Events; IHP, isolated hepatic perfusion; IPI, ipilimumab; MedDRA, Medical Dictionary for Regulatory Activities; NIVO, nivolumab.

a The denominator is variable among the treatment arms due to differences in exposure to the interventions preoperative IPI/NIVO, IHP and post-operative IPI/NIVO.

b Includes MedDRA terms arterial injury, procedural complication, procedural pain and wound dehiscence.

Eight patients in the post-operative arm received at least one cycle of post-operative IPI3/NIVO1; three of these patients (38%) had at least one grade 3 or 4 AE that was deemed by the investigators to be related to IPI3/NIVO1 (Table 2). Five patients (63%) discontinued IPI3/NIVO1 due to immune-related AEs (irAEs). Among the nine patients in the pre-post-operative arm who received one cycle of preoperative IPI3/NIVO1, two patients (22%) had at least one grade 3 or 4 AE that was deemed by the investigators to be related to IPI3/NIVO1. Of the eight patients in the pre-post-operative arm who received at least one cycle of post-operative IPI3/NIVO1, six (75%) had at least one grade 3 or 4 AE that was deemed by the investigators to be related to IPI3/NIVO1. In the pre-post-operative arm, 4/9 patients (45%) discontinued IPI3/NIVO1 due to irAEs.

Among patients who had at least one cycle of IPI3/NIVO1, 3/8 patients (38%) in the post-operative arm had an irAE of grade 3 or higher compared to 8/9 patients (89%) in the pre-post-operative arm, and the incidence of irAEs was numerically higher in the pre-post-operative approach (Supplementary Table S2, available at https://doi.org/10.1016/j.esmoop.2024.103623). A total of 20 SAEs, 10 in each arm, were reported in 11 patients (5/9 patients in the post-operative arm and 6/9 patients in the pre-post-operative arm; Supplementary Table S3, available at https://doi.org/10.1016/j.esmoop.2024.103623). There were no treatment-related deaths in either arm.

### Response

**Figure 2:**
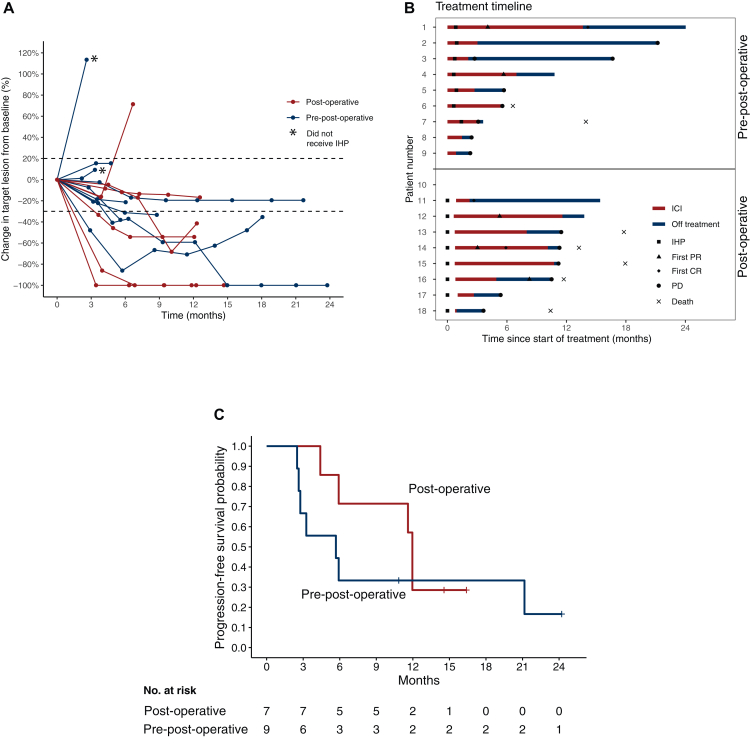
**Response, treatment and patient outcomes.** (A) Change in target lesion size from baseline. (B) Treatment and response timeline. (C) Progression-free survival. Tick marks indicate censored data. CR, complete response; ICI, immune checkpoint inhibitor; IHP, isolated hepatic perfusion; PD, progressive disease; PR, partial response.

Among the seven assessable patients in the post-operative arm, four patients (57%) had an objective response, including two patients (29%) with a CR (Table 3). Among the nine patients in the pre-post-operative arm, two patients (22%) had an objective response, including one patient (11%) with a CR. The difference in ORR was not statistically significant (*P* = 0.3, Fisher’s exact test). CBR, defined as patients who had a best response of CR, PR or SD, was 7/7 patients (100%) in the post-operative arm and 6/9 patients (67%) in the pre-post-operative arm. Temporal change in the target lesion diameter from baseline is shown in Figure 2A and a swimmer plot of treatment and response timelines is shown in Figure 2B.

**Table 3:** Best overall response as assessed by RECIST 1.1

	Post-operative arm^a^*n* (%)	Pre-post-operative arm *n* (%)	Total *n* (%)
Complete response	2 (22)	1 (11)	3 (17)
Partial response	2 (22)	1 (11)	3 (22)
Stable disease	3 (33)	4 (44)	7 (39)
Progressive disease	0 (0)	3 (33)	3 (17)
Overall response rate	4/7 (57)	2/9 (22)	6/16 (38)

a Two of the nine included patients in the post-operative arm were not assessable for efficacy.

At the time of data cut-off, response was maintained in 4/6 patients with a response (Supplementary Table S4, available at https://doi.org/10.1016/j.esmoop.2024.103623), with a median duration of response of 8.5 months (95% CI 8.5-20.6 months). Of note, one patient in the pre-post-operative arm was classified as having PD according to RECIST 1.1 criteria due to a new lesion at first radiological evaluation, which subsequently disappeared. According to immune-related RECIST, this patient was classified as having PR, lasting for 18 months before progression of disease.

### Progression-free survival

For the overall population (*n* = 16), the median PFS was 6.0 months (95% CI 0-12.4 months), but was longer when patients who did not receive IHP were excluded (median PFS 11.8 months, 95% CI 0-27.8 months, *n* = 14). PFS was numerically longer in the post-operative arm (11.8 months, 95% CI 8.0-15.6 months, *n* = 7) than in the pre-post-operative arm (6.0 months, 95% CI 5.5-6.5 months, *n* = 9) (Figure 2C). In the post-operative arm, there was one patient with extrahepatic disease at baseline who had simultaneous hepatic and extrahepatic progression. In the pre-post-operative arm, there were four patients with hepatic and extrahepatic disease at baseline, three of whom had progression at data cut-off, one due to hepatic progression, one due to extrahepatic progression and one due to simultaneous hepatic and extrahepatic progression.

## Discussion

In this phase Ib trial in patients with liver metastases of UM, a one-time treatment with IHP with melphalan combined with four cycles of IPI3/NIVO1 post-operatively resulted in an ORR of 57%. The study also compared this post-operative approach with a pre-post-operative approach, where patients received one cycle of IPI3/NIVO1 before IHP and then three additional cycles of IPI3/NIVO1. The hypothesis was that the pre-post-operative approach could increase efficacy at the risk of a higher toxicity rate. The results confirmed that the post-operative approach was associated with a lower rate of toxicity, but interestingly also a numerically higher ORR (57% versus 22%) and longer PFS (11.8 versus 6.0 months) than the pre-post-operative approach. However, these effects were associated with high rates of AEs for this combination treatment, higher than what historically has been described for the individual treatments.

In the pre-post-operative arm, 2/9 (22%) patients did not receive IHP as planned—one patient due to encephalitis following preoperative administration of IPI3/NIVO1 and the other patient due to a perioperative finding of extensive liver metastasis, possibly due to the delay of preoperative treatment before IHP. This frequency of dropout is higher than in the SCANDIUM trial of IHP,^19^ where 41/43 (95%) treatment-naïve patients received IHP as planned. In the post-operative arm, one patient did not complete the IHP procedure due to intraoperative damage to the hepatic artery. In a similar study, the CHOPIN trial investigating the combination of IPI1/NIVO3 and percutaneous hepatic perfusion (PHP), all patients received at least one cycle of PHP which thus seems like a potentially more tolerable combination.^20^ In this study, treatment discontinuation due to toxicity of IPI3/NIVO1 was 63% in the post-operative arm and 45% in the pre-post-operative arm, which is higher than in previous studies of patients with metastatic UM treated with IPI3/NIVO1 alone (23%-26%).^3^^,^^4^ Taken together, treatment with IHP and post-operative IPI3/NIVO1 was judged to have significant toxicity and treatment discontinuation, particularly in the pre-post-operative arm. However, it is worth noting that we observed durable responses in patients who received only one cycle of IPI3/NIVO1, which is in general agreement with previous studies on number of treatment cycles with IPI and NIVO, where the initial two cycles seem to have a major role for outcome.^21^, ^22^, ^23^

The incidence of treatment-related AEs attributed to IHP was similar in the post-operative and the pre-post-operative arms, but higher than previously reported for IHP alone, both in terms of grade >3 treatment-related AEs and SAEs.^9^^,^^24^ The proportion of grade 3 hepatitis in the post-operative arm was 67% compared to 33% in the pre-post-operative arm. The reason for this difference is not clear as we had anticipated a higher risk for immune activation and hepatitis in the pre-post-operative arm. There were three patients with grade 3 artery complications, and one patient with liver artery thrombosis detected 2 days after IHP in the post-operative arm. The patient was treated with low-molecular-weight heparin therapy but ultimately developed secondary biloma. Two patients had peroperative damage to the hepatic artery that was surgically resolved during the intervention; however, one of these patients could not receive IHP due to the complication. This frequency of 3/15 patients with vascular complications after IHP is higher than previously reported,^9^ without any obvious reasons. The incidence of grade 3 irAEs was 3/9 (33%) in the post-operative arm which is within the same range as previously reported for IPI/NIVO in the absence of IHP.^3^^,^^4^ Among patients in the pre-post-operative arm, 8/9 patients (89%) had a grade 3 or higher irAE, which was substantially higher than expected. The distribution of irAEs in relation to type and system organ class was diverse in both groups and without apparent differences between groups. However, we observed several rare and serious grade 3 or higher irAEs including two patients with encephalitis, one autoimmune hemolytic anemia, one pneumonitis, one colitis, one nephritis and three hepatitis cases. The irAEs were treated according to consensus guidelines for ICI toxicity. Although there were no treatment-related deaths, it is clear that irAEs are a clinical concern, particularly in the pre-post-operative approach. Nevertheless, given that only 18 patients were included in the trial, strong conclusions cannot be drawn concerning the distribution of toxicities and complications between the two study arms. The tolerability and toxicity concerns observed in this study suggest that modification to the treatment protocol to mitigate treatment discontinuation and toxicity is recommended, such as PHP with post-operative IPI1/NIVO3.

Of note, the recent approval of tebentafusp for the treatment of patients with metastatic UM and the HLA-A∗02:01 haplotype is a major advance.^6^ No patients in the present trial received tebentafusp since it was not available in Sweden during the inclusion phase. In the tebentafusp trial, the objective response rate was 9%, while several phase II trials of IPI and NIVO have reported ORRs ranging between 10% and 18%.^3^^,^^4^^,^^25^ In the SCANDIUM trial^19^ where treatment-naïve patients received IHP, the response rate was 40%, and in the FOCUS trial where patients received PHP, the response rate was 35%.^26^ In this trial, ORR was considerably higher, reaching 57% for patients who received post-operative IPI3/NIVO1 and IHP. Furthermore, the median PFS was 11.8 months among assessable patients in the current trial, compared to 3.3 months for tebentafusp,^6^ 7.4 months for IHP^19^ and 9.0 months for PHP.^26^

It is important to acknowledge the limitations inherent in this trial. Firstly, the relatively small sample size contributes to uncertainty in the outcomes, as reflected in the broad CIs observed. Additionally, the randomization process resulted in disparities between the groups in terms of prior treatments, M-stage and the presence of extrahepatic disease. These disparities are to suggest that the post-operative group may have exhibited a more favorable prognosis.

In summary, the combination of IHP and post-operative IPI3/NIVO1 in patients with liver-dominant metastases of UM was associated with a high rate of grade 3-4 AEs but without fatal events and with encouraging efficacy. A phase II trial comparing PHP with post-operative IPI1/NIVO3 compared with IPI3/NIVO1 is planned (the SCANDIUM III trial).
