# Supplementary material for: A phase Ib randomized multicenter trial of isolated hepatic perfusion in combination with ipilimumab and nivolumab for uveal melanoma metastases (SCANDIUM II trial)
Source: ESMO Open. 2024 Jul 2;9(7):103623. doi: 10.1016/j.esmoop.2024.103623 (PMC11269777; doi:10.1016/j.esmoop.2024.103623)
Supplement: Supplementary Table [file mmc1.docx]

**Supplementary Table S1.** Patients with grade 1-2 adverse events attributed by the treating physician to IHP or ipilimumab and nivolumab graded according to CTCAE 4.0 and classified according to MedDRA.

|  |  | **ARM** | | | | | | | |
| --- | --- | --- | --- | --- | --- | --- | --- | --- | --- |
|  |  | **Post-op** | | | | **Pre-Post-op** | | | |
|  |  | **IHP** | | **IPI‎/NIVO** | | **IHP** | | **IPI‎/NIVO** | |
|  | **Event** |  | |  | |  | |  | |
|  |  | **grade 1** | **grade 2** | **grade 1** | **grade 2** | **grade 1** | **grade 2** | **grade 1** | **grade 2** |
| Blood and lymphatic system disorders | Anaemia | 3 | 5 | 0 | 0 | 2 | 5 | 0 | 0 |
|  | Thrombocytopenia | 4 | 0 | 0 | 0 | 2 | 1 | 0 | 0 |
|  | Leukocytopenia | 0 | 0 | 0 | 0 | 2 | 0 | 0 | 0 |
|  | Leukocytosis | 3 | 0 | 1 | 0 | 5 | 0 | 0 | 0 |
|  | Lymph node pain | 0 | 0 | 0 | 1 | 0 | 0 | 0 | 0 |
| Cardiac disorders | Atrial fibrillation | 0 | 0 | 1 | 0 | 0 | 1 | 0 | 0 |
|  | Dizziness | 0 | 0 | 0 | 0 | 1 | 0 | 1 | 0 |
|  | Dyspnoea | 0 | 0 | 1 | 0 | 0 | 0 | 0 | 0 |
|  | Fluid overload | 0 | 0 | 0 | 0 | 0 | 1 | 0 | 0 |
|  | Oedema peripheral | 0 | 0 | 0 | 0 | 1 | 0 | 0 | 0 |
|  | Sinus tachycardia | 0 | 0 | 0 | 0 | 1 | 0 | 0 | 0 |
|  | Tachycardia | 3 | 0 | 0 | 0 | 2 | 0 | 0 | 0 |
| Endocrine disorders | Hypothyroidism | 0 | 0 | 1 | 0 | 0 | 0 | 1 | 0 |
|  | Hyperthyroidism | 0 | 0 | 0 | 0 | 0 | 0 | 1 | 0 |
| Gastrointestinal disorders | Abdominal distension | 0 | 0 | 0 | 0 | 1 | 0 | 0 | 0 |
|  | Abdominal hernia | 0 | 0 | 0 | 0 | 1 | 0 | 0 | 0 |
|  | Abdominal pain | 1 | 2 | 0 | 1 | 0 | 1 | 0 | 0 |
|  | Colitis | 0 | 0 | 0 | 1 | 0 | 0 | 0 | 0 |
|  | Constipation | 2 | 2 | 0 | 0 | 5 | 0 | 0 | 0 |
|  | Diarrhoea | 0 | 0 | 1 | 0 | 1 | 0 | 1 | 0 |
|  | Dry mouth | 0 | 0 | 0 | 1 | 0 | 0 | 1 | 0 |
|  | Gastritis | 0 | 0 | 0 | 1 | 0 | 0 | 0 | 1 |
|  | Nausea | 3 | 3 | 0 | 0 | 3 | 4 | 0 | 0 |
|  | Oral candidiasis | 0 | 0 | 0 | 0 | 1 | 0 | 0 | 0 |
|  | Stomatitis | 0 | 0 | 1 | 1 | 0 | 0 | 0 | 0 |
|  | Vomiting | 1 | 1 | 0 | 0 | 0 | 5 | 0 | 0 |
| General disorders and administration site conditions | Chills | 1 | 0 | 1 | 0 | 0 | 0 | 0 | 0 |
|  | Decreased appetite | 3 | 1 | 0 | 1 | 0 | 0 | 0 | 0 |
|  | Drug eruption | 0 | 0 | 1 | 0 | 0 | 0 | 0 | 0 |
|  | Fatigue | 1 | 2 | 2 | 0 | 0 | 1 | 5 | 1 |
|  | Influenza like illness | 0 | 1 | 0 | 0 | 0 | 0 | 0 | 2 |
|  | Infusion related reaction | 0 | 0 | 0 | 0 | 0 | 0 | 0 | 1 |
|  | Postoperative fever | 1 | 0 | 0 | 0 | 0 | 0 | 0 | 0 |
|  | Pyrexia | 9 | 0 | 0 | 1 | 5 | 2 | 1 | 0 |
| Hepatobiliary disorders | Hepatitis | 0 | 0 | 0 | 0 | 0 | 0 | 0 | 1 |
| Immune system disorders | Cutaneous sarcoidosis | 0 | 0 | 2 | 0 | 0 | 0 | 0 | 0 |
|  | Dermatomyositis | 0 | 0 | 0 | 1 | 0 | 0 | 0 | 0 |
| Infections and infestations | Rhinitis | 0 | 0 | 0 | 0 | 0 | 0 | 1 | 0 |
|  | Skin infection | 0 | 1 | 0 | 0 | 0 | 0 | 0 | 0 |
|  | Wound infection bacterial | 0 | 1 | 0 | 0 | 0 | 0 | 0 | 0 |
| Injury, poisoning and procedural complications | Procedural pain | 0 | 2 | 0 | 0 | 1 | 3 | 0 | 0 |
|  | Urinary retention postoperative | 0 | 1 | 0 | 0 | 0 | 0 | 0 | 0 |
| Investigations | Activated partial thromboplastin time prolonged | 0 | 0 | 0 | 0 | 1 | 0 | 0 | 0 |
|  | Alanine aminotransferase increased | 1 | 2 | 0 | 1 | 3 | 1 | 2 | 1 |
|  | Aspartate aminotransferase increased | 1 | 2 | 0 | 0 | 2 | 1 | 2 | 2 |
|  | Blood albumin decreased | 8 | 0 | 0 | 0 | 2 | 1 | 0 | 0 |
|  | Blood alkaline phosphatase decreased | 1 | 0 | 1 | 0 | 2 | 0 | 0 | 0 |
|  | Blood alkaline phosphatase increased | 4 | 0 | 0 | 0 | 2 | 1 | 0 | 1 |
|  | Blood bilirubin increased | 1 | 1 | 0 | 0 | 1 | 0 | 0 | 0 |
|  | Blood calcium decreased | 0 | 0 | 0 | 0 | 1 | 0 | 0 | 0 |
|  | Blood creatinine increased | 1 | 0 | 0 | 0 | 0 | 0 | 0 | 0 |
|  | Blood glucose increased | 0 | 0 | 0 | 0 | 1 | 0 | 0 | 0 |
|  | Blood lactate dehydrogenase increased | 1 | 0 | 0 | 0 | 1 | 0 | 1 | 0 |
|  | Blood potassium decreased | 1 | 0 | 0 | 0 | 2 | 0 | 0 | 0 |
|  | Blood potassium increased | 1 | 0 | 0 | 0 | 1 | 0 | 0 | 0 |
|  | Blood sodium decreased | 2 | 0 | 0 | 0 | 2 | 0 | 0 | 0 |
|  | Blood urea increased | 0 | 0 | 0 | 0 | 0 | 1 | 0 | 0 |
|  | Blood uric acid decreased | 1 | 0 | 0 | 0 | 1 | 0 | 0 | 0 |
|  | Blood uric acid increased | 0 | 0 | 0 | 0 | 1 | 0 | 0 | 0 |
|  | C-reactive protein increased | 7 | 1 | 0 | 0 | 2 | 2 | 0 | 0 |
|  | Cortisol decreased | 0 | 0 | 0 | 0 | 0 | 0 | 1 | 0 |
|  | Glomerular filtration rate | 2 | 0 | 0 | 0 | 1 | 0 | 0 | 0 |
|  | International normalised ratio increased | 2 | 0 | 0 | 0 | 1 | 1 | 0 | 0 |
|  | Myoglobin blood increased | 2 | 0 | 0 | 0 | 2 | 0 | 0 | 0 |
|  | Prothrombin level increased | 2 | 0 | 0 | 0 | 0 | 1 | 0 | 0 |
|  | Troponin increased | 0 | 0 | 1 | 0 | 0 | 0 | 0 | 0 |
|  | Urine output decreased | 1 | 0 | 0 | 0 | 0 | 0 | 0 | 0 |
|  | Weight decreased | 0 | 0 | 0 | 0 | 2 | 0 | 0 | 0 |
| Musculoskeletal and connectiv tissue disorders | Arthralgia | 0 | 0 | 1 | 3 | 1 | 0 | 0 | 0 |
|  | Myalgia | 0 | 0 | 1 | 0 | 1 | 0 | 1 | 0 |
| Nervous system disorders | Headache | 1 | 0 | 0 | 0 | 0 | 0 | 2 | 0 |
|  | Insomnia | 0 | 0 | 0 | 0 | 1 | 0 | 0 | 0 |
|  | Paraesthesia | 0 | 0 | 1 | 0 | 0 | 0 | 0 | 0 |
| Psychiatric disorders | Anxiety | 0 | 0 | 0 | 0 | 0 | 1 | 0 | 0 |
| Renal and urinary disorders | Polyuria | 2 | 0 | 0 | 0 | 1 | 1 | 0 | 0 |
| Respiratory, thoracic and mediastinal disorders | Cough | 0 | 0 | 0 | 0 | 1 | 0 | 0 | 0 |
|  | Pleural effusion | 0 | 1 | 0 | 0 | 0 | 0 | 0 | 0 |
|  | Hypoxia | 3 | 1 | 0 | 0 | 0 | 1 | 0 | 0 |
|  | Pneumonitis | 0 | 0 | 1 | 0 | 0 | 0 | 0 | 0 |
|  | Pulmonary embolism | 0 | 1 | 0 | 0 | 0 | 0 | 0 | 0 |
| Skin and subcutaneous tissue disorders | Dry skin | 0 | 0 | 0 | 0 | 0 | 0 | 1 | 0 |
|  | Pruritus | 3 | 0 | 2 | 0 | 1 | 0 | 2 | 0 |
|  | Rash | 0 | 0 | 2 | 0 | 2 | 1 | 1 | 2 |
|  | Urticaria | 0 | 0 | 0 | 0 | 1 | 0 | 0 | 1 |
| Vascular disorders | Haematoma | 0 | 0 | 0 | 0 | 1 | 0 | 0 | 0 |
|  | Hypotension | 2 | 1 | 0 | 0 | 1 | 1 | 0 | 0 |

**Supplementary Table S2.** Total number of treatment-related adverse events according to CTCAE v4.0

|  | **Post op** | | **Pre-post op** | |
| --- | --- | --- | --- | --- |
| **Grade** | **IHP** | **IPI/NIVO** | **IHP** | **IPI/NIVO** |
| **1** | 87 | 24 | 81 | 44 |
| **2** | 34 | 15 | 42 | 25 |
| **3** | 19 | 8 | 20 | 16 |
| **4** | 3 | 1 | 1 | 1 |
| **Total** | **143** | **48** | **144** | **86** |

Abbreviations: IHP, isolated hepatic perfusion; IPI, ipilimumab; NIVO, nivolumab

**Supplementary Table S3.** Serious adverse events in relation to study intervention

| **Arm** | **MedDRA term** | **AE grade** | **Relation to study intervention*** |
| --- | --- | --- | --- |
| **Post op** | Artery dissection | 3 | IHP |
|  | Arterial thrombosis | 3 | IHP |
|  | Intraoperative hepatobiliary injury | 3 | IHP |
|  | Wound infection bacterial | 2 | IHP |
|  | Intraoperative arterial injury | 4 | IHP |
|  | Encephalitis | 3 | IPI/NIVO |
|  | Autoimmune hemolytic anemia | 4 | IPI/NIVO |
|  | Colitis | 3 | IPI/NIVO |
|  | Colitis | 3 | IPI/NIVO |
|  | Diarrhoea, *Clostridium difficile* | 3 | Not related |
| **Pre-post op** | Platelet count decreased | 2 | IHP |
|  | Pleural effusion | 3 | IHP |
|  | Nephritis | 3 | IHP/IPI/NIVO |
|  | Fever | 3 | IHP/IPI/NIVO |
|  | Hepatitis | 2 | IHP/IPI/NIVO |
|  | Pneumonitis | 3 | IPI/NIVO |
|  | Encephalitis | 3 | IPI/NIVO |
|  | Diabetes mellitus | 4 | IPI/NIVO |
|  | Fever | 3 | IPI/NIVO |
|  | Infection UNS | 3 | Not related |

*As assessed by the treating physician

Abbreviations: AE, adverse event; IHP, isolated hepatic perfusion; IPI, ipilimumab; NIVO, nivolumab; UNS, unspecified

**Supplementary Table S4.** Time to response (TTR) and duration of response (DOR) in months as assessed by RECIST 1.1

| **ID** | **Arm** | **BOR** | **TTR** | **DOR** | **Ongoing** |
| --- | --- | --- | --- | --- | --- |
| 104 | Post op | CR | 4 | 8 | No |
| 106 | Post op | CR | 4 | 13+ | Yes |
| 303 | Post op | PR | 10 | 2 | No |
| 304 | Post op | PR | 6 | 9+ | Yes |
| 108 | Pre-post op | PR | 6 | 5+ | Yes |
| 301 | Pre-post op | CR | 5 | 20+ | Yes |

Abbreviations: BOR, best overall response; TTR, time to response; DOR, duration of response; CR, complete response; PR, partial response
